# Low Stroke Volume Predicts Deterioration in Intermediate-Risk Pulmonary Embolism: Prospective Study

**DOI:** 10.5811/westjem.18434

**Published:** 2024-06-14

**Authors:** Anthony J. Weekes, Parker Hambright, Ariana Trautmann, Shane Ali, Angela Pikus, Nicole Wellinsky, Sanjeev Shah, Nathaniel O’Connell

**Affiliations:** * Atrium Health’s Carolinas Medical Center, Department of Emergency Medicine, Charlotte, North Carolina; † Atrium Health Sanger Heart and Vascular Institute, Charlotte, North Carolina; ‡ Wake Forest University School of Medicine, Department of Biostatistics and Data Science, Winston-Salem, North Carolina

## Abstract

**Introduction:**

Prognosis and management of patients with intermediate-risk pulmonary embolism (PE) is challenging. We investigated whether stroke volume may be used to identify the subset of this population at increased risk of clinical deterioration or PE-related death. Our secondary objective was to compare echocardiographic measurements of patients who received escalated interventions vs anticoagulation monotherapy.

**Methods:**

We selected patients with intermediate-risk PE, who had comprehensive echocardiography within 18 hours of PE diagnosis and before any escalated interventions, from a PE registry populated by 11 emergency departments. Echocardiographers measured right ventricle (RV) size, tricuspid annular plane systolic excursion (TAPSE), and stroke volume (SV) using velocity time integral (VTI) by left ventricular (LV) outflow tract Doppler or two-dimensional method of discs (MOD). The primary outcome was a composite of PE-related death, cardiac arrest, catecholamine administration for sustained hypotension, or emergency respiratory intervention during the index hospitalization. Secondary outcome was escalated intervention with reperfusion or extracorporeal membrane oxygenation therapy.

**Results:**

Of 370 intermediate-risk PE patients (mean age 64.0 ± 15.5 years, 38.1% male), 39 (10.5%) had the primary outcome. These 39 patients had lower mean SV regardless of measurement method than those without the primary outcome: SV MOD 36.2 vs 49.9 milliliters (mL), *P* < 0.001; SV Doppler 41.7 vs 57.2 mL, *P* = 0.003; VTI 13.6 vs 17.9 centimeters [cm], *P* = 0.003. Patients with primary outcome also had lower mean TAPSE than those without (1.54 vs 1.81 cm, *P* = 0.003). Multivariable models, selecting SV as predictor, had area under the receiver operating curve of 0.8 and Brier score 0.08. The best echocardiographic predictor of our primary outcome was SV MOD (odds ratio 0.72 [0.53, 0.94], *P* = 0.02). Patients who received escalated interventions had significantly lower SV or surrogate measurements, greater RV dilatation, and lower RV systolic function than patients who received anticoagulation monotherapy.

**Conclusion:**

Low stroke volume was a predictor of clinical deterioration and PE-related death. Low SV may be used to identify a subset of intermediate-risk PE patients, who are higher risk (intermediate-high risk), and for whom escalated interventions should be considered.

Population Health Research Capsule What do we already know about this issue?

*Right ventricle dysfunction identifies intermediate-risk pulmonary embolism (PE) but may not predict increased likelihood of hemodynamic instability.*

What was the research question?

*Do hemodynamic parameters such as stroke volume (SV) predict clinical deterioration in intermediate-risk PE?*

What was the major finding of the study?

*A predictive model for clinical deterioration in PE patients including stroke volume had AUC 0.81 (95% CI 0.69, 0.92) and Brier score 0.08 (0.06, 0.10).*

How does this improve population health?

*Low stroke volume may identify intermediate-high risk PE, ie, those at greater risk for clinical deterioration and death.*

## INTRODUCTION

Pulmonary embolism (PE) risk stratification tools focus on presence or absence of right ventricle (RV) dysfunction and hemodynamic stability.^1^^–^^5^ Patients with PE who have RV dysfunction and are hemodynamically stable are classified as intermediate risk (submassive) by the European Society of Cardiology (ESC) and CHEST guidelines.^1^^,^^5^^–^^8^ However, there is a spectrum of disease severity within this classification. While most intermediate-risk patients improve with anticoagulation only, some may need more intensive inpatient monitoring and escalated interventions due to acute clinical deterioration. The challenge is to identify which intermediate-risk patients are at the higher end of the risk spectrum.

Those who are at greater risk for hemodynamic instability or clinical deterioration are classified as ***intermediate-high*** ***risk*** (severe submassive) by the ESC and CHEST. This subset is defined by troponin elevation with ESC guidelines; however, this strategy has low positive predictive value.^1^^,^^5^^,^^9^^,^^10^ While some PE response teams (PERT) use ESC guidelines, others use clinical signs of hypoxia, episodic hypotension, or elevated shock index to identify intermediate-high risk PE. How intermediate-high risk is classified matters because physician decisions regarding escalated treatments are based on the predicted risk of acute clinical deterioration.

Expert researchers argue that qualitative RV dilatation is insufficient to identify patients suffering from a low-flow state and likely to experience clinical deterioration.^8^^,^^9^^,^^11^ It is physiologically plausible that inadequate left ventricle (LV) filling with reduced stroke volume (SV) may signal more severe PE within the intermediate-risk group than RV dilatation or elevated laboratory measurements of myocardial injury.^12^ Reduced SV, a hemodynamic parameter, may identify those at increased risk for acute clinical deterioration (defined herein as cardiac arrest, catecholamine administration for sustained hypotension, or emergency respiratory intervention during the index hospitalization) or PE-related death. The PE literature, however, rarely reports on SV for risk stratification or prognosis of acute clinical deterioration.^6^^,^^7^^,^^11^^–^^14^

Our primary objective was to compare prognostic performance of SV measurements in comparison to RV measurements to characterize the relationship between echocardiographic hemodynamic parameters, including SV, and acute clinical deterioration in emergency department (ED) patients classified as intermediate-risk PE. We hypothesized that those who experienced clinical deterioration would have lower SV at presentation than those who did not. Our secondary objective was to compare initial echocardiographic measurements of patients who received escalated interventions with those who received anticoagulation monotherapy. We hypothesized initial SV measurements would be significantly different between treatment groups.

## METHODS

### Study Design and Settings

We identified patients from our prospective, observational Clinical Outcomes in Pulmonary Embolism Research Registry (COPERR). The COPERR was populated with patients diagnosed with intermediate- or high-risk PE in any of our health system’s 11 EDs between June 2018–August 2022. All COPERR patients had confirmed acute PE with RV to LV basal diameter ratio (RV:LV) ≥ 1.0 by computed tomography (CT) or point-of-care echocardiography, or cardiac biomarker elevation (brain natriuretic peptide [BNP], troponin, or high sensitivity troponin). We used the 2019 ESC PE guidelines to classify COPERR patients as high risk and intermediate-low risk PE^5^; however, we used an institution-specific definition for intermediate-high risk (which was informed by the 2019 ESC guidelines).^5^ We classified patients as intermediate-high risk if they had RV dilatation and one or more of the following signs: episodic hypotension (systolic blood pressure [SBP] 90 millimeters of mercury [mm Hg] <15 minutes); sustained shock index >1.0; or pulse oximetry reading <92% on room air with respiratory distress. For the registry, board-certified radiologists reviewed CT images and reported RV dilatation, and sonographers performed comprehensive transthoracic echocardiography (TTE).

The Atrium Health Institutional Review Board approved COPERR and planned analyses (including this study) with a waiver of informed consent. Clinicians were blind to study design and hypothesis and managed patients without guidance or recommendations.

### Subjects

We included COPERR patients classified as intermediate risk at ED presentation, who had TTE within 18 hours of PE order set being placed and before any escalated interventions. Atrium Health has a multidisciplinary PERT equipped with an intermediate- and high-risk PE order set within the electronic health record (EHR). The TTE can be ordered separately or as part of the PE order set. Most patients with PERT activations had TTE pre-ordered as part of the PE order set. We excluded patients if any of the following criteria were present: 1) PE was incidental finding on imaging; 2) PE was not the primary diagnosis contributing to patient’s clinical presentation to the ED; 3) PE diagnosis secured >2 hours after admission from the ED; 4) non-acute PE with similar filling defects (unchanged or resolving) if previous CT available; 5) hemodynamic instability attributable to PE, including sustained hypotension (SBP below 90 mm Hg >15 minutes) or unstable cardiac rhythms or obstructive shock or cardiac arrest (classification as high risk)^5^; 6) TTE was not completed or was without RV or SV measurements; and 7) escalated intervention performed before TTE.

### Data Collection

Data extractors were trained in the explanation of all variables and identification of EHR source documents. Those who completed successful trials of data extraction on test cases were qualified to monitor the EHR for study data entry into Research Electronic Data Capture (REDCap, hosted at Atrium Health’s Carolinas Medical Center) case report forms, which had detailed field notes to enhance reliability.^15^ Extractors who retrieved echocardiography measurements were blind to patient outcomes. A project manager monitored data accuracy and completeness.

### Measurements

#### Cardiac Biomarkers

Samples and measurements were obtained while patients were in the ED. We used an i-STAT cardiac troponin test cartridge (Abbott Laboratories, Abbott Park, IL), measured in nanograms per milliliter (ng/mL) for troponin I or high-sensitivity troponin assays. Normal values for troponin I were < 0.07 ng/mL. Normal values for high-sensitivity troponin were <12 for females and <20 for males. We used the i-STAT BNP test cartridge (Abbott) measured in picograms (pg)/mL. Normal point-of-care BNP measurements were 90 ng/mL.

#### Transthoracic Echocardiography

Trained sonographers (blind to research study and patient outcomes) performed TTE measurements following the American Society of Echocardiography guidelines^16^^,^^17^ at an echocardiography facility accredited by the Intersocietal Commission for the Accreditation of Echocardiography Laboratories. TTE was completed and recorded before the primary outcome or any escalated interventions occurred. Measurements included chamber dimensions and systolic function for left and right ventricles and left ventricular SV. Digital images and video were mapped from echocardiography machines and stored in Merge Cardio (Merative LP, Ann Arbor, MI), an imaging archiving platform. The cardiologist-investigator (blind to patient presentation and outcomes) reviewed ventricular and SV measurements or performed de novo two-dimensional (2D) measurements on the imaging platform.

#### Ventricular Chamber Size

We used apical 4-chamber or RV focused apical view to measure end-diastolic internal measurements of the RV in short axis (mid and basal levels) and long axis (length). We used parasternal long axis view to measure LV basal diameter. We calculated the RV:LV basal diameter ratio.

#### Right Ventricle Systolic Function

In the apical view, we used M mode to measure tricuspid annular plane systolic excursion (TAPSE) of the RV free wall tricuspid annulus. We used tissue Doppler to measure peak systolic velocity of the basal RV free wall segment and continuous wave Doppler to measure peak tricuspid regurgitation velocity during systole and to estimate right atrial pressure. Trace or unmeasurable regurgitation velocities were categorized as a discrete response rather than considered missing.

#### Cardiac Output

We calculated cardiac output (CO) as SV multiplied by heart rate. (The SV is often used as a surrogate of CO.^18^^,^^19^) We calculated SV from the LV by 2D method of discs (MOD) or pulsed wave Doppler.^19^ In patients who had pulsed wave Doppler tracings recorded, we calculated SV by using left ventricular outflow tract (LVOT) diameter taken in the parasternal long axis and multiplying LVOT area by velocity time integral (VTI) of LVOT using the apical 5-chamber view. The VTI may be used as a surrogate of SV.^20^^,^^21^ When available, a biplane MOD was also used for apical-4 and apical-2 chamber views to calculate differences between end-diastolic and end-systolic volume. When only an apical-4 chamber view was available, we used MOD. When both views were available, the average of apical-4 and -2 SV measurements was used.

Because 2D methods do not account for mitral regurgitant flow, we reported absence or presence of mitral regurgitation (MR). If present, MR was graded as mild, moderate, or severe. Body surface area (BSA) was available for indexing of measurements.

### Outcomes

The primary outcome was a composite of PE-related death or clinical deterioration, defined as cardiac arrest, catecholamine administration for sustained hypotension, or emergency respiratory intervention during the index hospitalization. The secondary outcome was use of one or more escalated interventions, including reperfusion interventions (systemic thrombolysis [full or reduced dose]), catheter-directed interventions, advanced endovascular interventions, surgical thrombectomy, and extracorporeal membrane oxygenation [ECMO]).

### Statistical Analyses

Study sample was determined by the number of registry patients eligible for inclusion. Analyses specific to each objective follow. We used R software (R Project for Statistical Computing, Vienna, Austria) for all analyses.

#### Primary Objective

For TTE variables, we reported the number of observations, means with standard deviations, or frequencies. We compared differences in means between primary outcome groups using unpaired *t*-tests for continuous variables and chi-square tests for categorical variables. We reported the percentage of missing observations for each variable and used imputation for multivariable analyses. We performed bivariable and multivariable logistic regression to assess associations of echocardiographic measurements with the primary outcome. For patients with SV measured by both Doppler and MOD methods, we determined two-sided 95% confidence intervals (CI) for the Pearson correlation coefficient between SV measurements. Because SV and CO are inherently correlated, including both within the same multivariable model would lead to multicollinearity and variance inflation. Therefore, we fit two separate models for each outcome, one with Doppler-derived SV or CO as a predictor, and a second one with MOD-derived SV or CO as a predictor. Each model contained the same other predictors.

We fit multivariable logistic regression models for our primary outcome, including TTE and non-TTE measurements independently associated with the primary outcome in the univariable models (*P* < 0.10). We fit a multivariable logistic regression model for our primary composite outcome. To select the best fitting model while controlling for key sources of confounding and issues with multicollinearity between clinical predictors of interest, we used least absolute shrinkage and selection operator (LASSO) regression with 10-fold cross validation to select our final logistic regression model. The SV MOD, SV LVOT, CO MOD, and CO LVOT all induced variance inflation due to collinearity when included in the same model. From univariable bivariable logistic models, we determined optimal thresholds for predicting our primary outcome for each TTE metric using Youden’s J-statistic.^22^^,^^23^ We reported performance metrics of these thresholds as sensitivity, specificity, positive predictive value (PPV), negative predictive value (NPV), and odds ratios (OR) with 95% CIs for predicting clinical deterioration.

We used the full dataset to fit a random forest (RF) model. We generated a variable importance plot based on mean decrease in accuracy to assess importance of predictors and compare them with the significance of univariable bivariable associations based on *t*-tests. We reported prognostic performance of LASSO and RF with AUC, Brier score, scaled Brier score, calibration intercept, slope, and plot. Finally, to address potential inaccuracies of predicted probabilities with unbalanced data or translation into clinical utility, we reported on net benefit based on decision curve analysis.^27^^,^^28^

#### Secondary Objective

We compared echocardiographic measurements between groups that received anticoagulation monotherapy vs escalated interventions with the unpaired *t*-test.

## RESULTS

Of 370 patients who met inclusion criteria, 363 (98.1%) were seen July 2020–August 2022; four patients were from 2018; and three patients from 2019 (Figure). There were no significant differences in demographics between outcome groups (Table 1). Patients with primary outcome had higher respiratory and heart rates at presentation and lower SBP and oxygen saturation than those without. Initial high-sensitivity troponin elevation was not significantly different between primary outcome groups.

**Figure.**
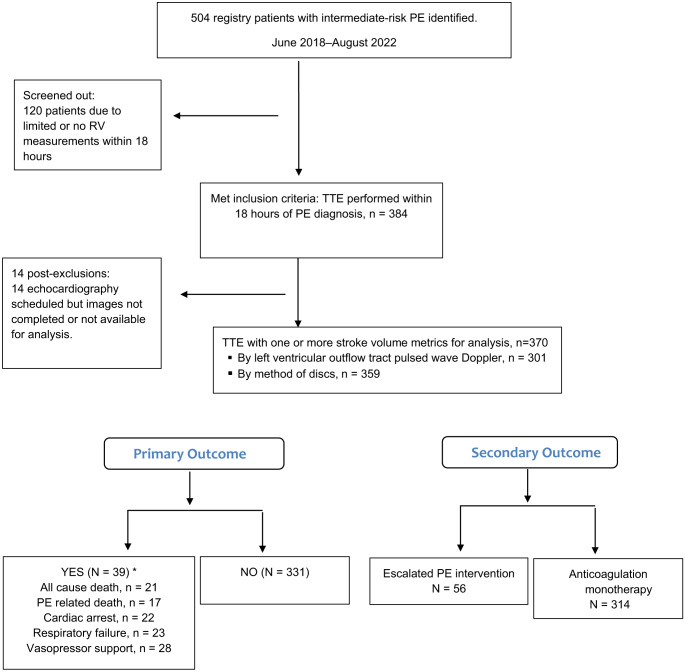
Screening and patient flow diagram. *PE*, pulmonary embolism; *RV*, right ventricle; *TTE*, transthoracic echocardiography. *Components of the primary composite outcome are not mutually exclusive.

**Table 1.** Patient characteristics and clinical presentation by primary outcome.

Patient characteristics	Primary outcome = YES (N = 39)	Primary outcome = NO (N = 331)	Overall (N = 370)	*P*-value
**Age, years**				
Mean (SD)	66.4 (13.5)	62.3 (16.0)	62.7 (15.8)	0.12
Missing	0 (0%)	1 (0.3%)	1 (0.3%)	
**Race**				
African-American	16 (41.0%)	123 (37.4%)	139 (37.6%)	0.36
Other	3 (7.7%)	13 (4.0%)	16 (4.3%)	
White	20 (51.3%)	193 (58.7%)	215 (58.1%)	
**Ethnicity**				
Hispanic	2 (5.1%)	14 (4.3%)	16 (4.3%)	0.77
Non-Hispanic	37 (94.9%)	312 (94.8%)	351 (94.9%)	
Unknown	0 (0%)	3 (0.9%)	3 (0.8%)	
**Gender**				
Female	17 (43.6%)	173 (52.6%)	192 (51.9%)	0.31
Male	22 (56.4%)	156 (47.4%)	178 (48.1%)	
**Lowest systolic blood pressure within 3 hours of presentation (mmHg)**				
Mean (SD)	97.8 (30.7)	122 (23.8)	120 (25.7)	<0.001
**Highest heart rate within 3 hours of presentation (beats per minute)**				
Mean (SD)	122 (21.1)	107 (22.1)	108 (22.5)	<0.001
**Lowest oxygen saturation on room air within 3 hours of presentation (%)**				
Mean (SD)	86.6 (15.5)	93.6 (5.34)	92.8 (7.40)	<0.001
**Highest respiratory rate within 3 hours of presentation (breaths per minute)**				
Mean (SD)	32.5 (13.0)	25.2 (9.17)	26.0 (9.88)	<0.001
**Body surface area, m2**				
Mean (SD)	1.94 (0.25)	2.08 (0.31)	2.07 (0.31)	0.01
Missing	4 (10.3%)	24 (7.3%)	29 (7.8%)	
**Dementia**				
No	34 (87.2%)	314 (94.9%)	348 (94.1%)	0.06
Yes	5 (12.8%)	16 (4.9%)	21 (5.7%)	
Missing	0 (0%)	1 (0.3%)	1 (0.3%)	
**Chronic obstructive pulmonary disease**				
No	34 (87.2%)	288 (87.0%)	322 (87.0%)	0.99
Yes	5 (12.8%)	42 (12.7%)	47 (12.7%)	
Missing	0 (0%)	1 (0.3%)	1 (0.3%)	
**Metastatic solid tumor**				
No	30 (76.9%)	310 (93.6%)	340 (91.9%)	0.001
Yes	9 (23.1%)	20 (6.1%)	29 (7.8%)	
Missing	0 (0%)	1 (0.3%)	1 (0.3%)	
**Any malignancy**				
No	33 (84.6%)	285 (86.1%)	318 (85.9%)	0.80
Yes	6 (15.4%)	45 (13.6%)	51 (13.8%)	
Missing	0 (0%)	1 (0.3%)	1 (0.3%)	
**Prior diagnosis of pulmonary embolism**				
No	32 (82.1%)	248 (74.9%)	280 (75.7%)	0.43
Yes	7 (17.9%)	82 (24.9%)	89 (24.1%)	
Missing	0 (0%)	1 (0.3%)	1 (0.3%)	
**Recent hospitalization**				
No	26 (66.7%)	290 (87.6%)	316 (85.4%)	0.001
Yes	13 (33.3%)	40 (12.1%)	53 (14.3%)	
Missing	0 (0%)	1 (0.3%)	1 (0.3%)	
**Current prescribed anticoagulation**				
No	36 (92.3%)	295 (89.1%)	331 (89.5%)	0.78
Yes	3 (7.7%)	35 (10.6%)	38 (10.3%)	
Missing	0 (0%)	1 (0.3%)	1 (0.3%)	
**Recent trauma**				
No	39.0 (100%)	315 (95.2%)	354 (95.7%)	0.39
Yes	0 (0%)	15 (4.5%)	15 (4.1%)	
Missing	0 (0%)	1 (0.3%)	1 (0.3%)	
**Family history of venous thromboembolism**				
No	39 (100%)	308 (93.1%)	347 (93.8%)	0.24
Yes	0 (0%)	18 (5.4%)	18 (4.9%)	
Missing	0 (0%)	5 (1.5%)	5 (1.4%)	
**Hormonal replacement therapy**				
No	38 (97.4%)	315 (95.2%)	353 (95.4%)	1.00
Yes	1 (2.6%)	15 (4.5%)	16 (4.3%)	
Missing	0 (0%)	1 (0.3%)	1 (0.3%)	
**Tobacco use**				
Current	9 (23.1%)	65 (19.6%)	74 (20.0%)	0.16
Ex smoker (smoked >100 cigarettes in their lifetime but has not smoked in the last 28 days but less than 12 months)	4 (10.3%)	11 (3.3%)	15 (4.1%)	
Ex smoker for >12 months	4 (10.3%)	56(16.9%)	60 (16.2%)	
Never	22 (56.4%)	197 (59.5%)	219 (59.2%)	
Missing	0 (0%)	2 (0.6%)	2 (0.5%)	
**Severe renal disease**				
No	35 (89.7%)	293 (88.5%)	328 (88.6%)	1.00
Yes	4 (10.3%)	37 (11.2%)	41 (11.1%)	
Missing	0 (0%)	1 (0.3%)	1 (0.3%)	
**Congestive heart failure**				
No	33 (84.6%)	297 (89.7%)	330 (89.2%)	0.27
Yes	6 (15.4%)	33 (10%)	39 (10.5%)	
Missing	0 (0%)	1 (0.3%)	1 (0.3%)	
**Hemi- or paraplegia**				
No	38 (97.4%)	323 (97.6%)	361 (97.6%)	0.60
Yes	1 (2.6%)	7 (2.1%)	8 (2.2%)	
Missing	0 (0%)	1 (0.3%)	1 (0.3%)	

As shown in Figure, 39 of 370 patients (10.5%) had the primary outcome. Of 21 (5.7%) patients who died, only 17 (4.6%) PE-related deaths were counted as having the primary outcome. The SV measurement was by LVOT Doppler method in 301 (81.4%) patients and by MOD in 359 (97.0%). In 290 patients, both SV measurement methods were used, with a correlation coefficient of 0.69 (0.63, 0.75). The CO had correlation coefficient of 0.66 (0.59, 0.72). Escalated interventions occurred in 56 (15.1%) patients, with 39 receiving systemic thrombolysis, 15 receiving catheter-directed intervention (CDI), two receiving ECMO, and one receiving surgical embolectomy. One patient had both systemic thrombolysis and CDI. Of 15 patients receiving CDIs, 12 had catheter-directed thrombolysis (10 ultrasound-assisted and two non-ultrasound assisted), and four had aspiration thrombectomy (data not shown).

Table 2 shows that both Doppler- and MOD-derived output measurements were lower for those with than without the primary outcome. In contrast, for RV systolic function, mean TAPSE was lower (worse) in those with than without the primary outcome. Most values were lower than mean values for a healthy cohort (Table 2 footnote). There were no significant differences in TTE metrics for RV size, with only RV:LV ratio approaching statistical significance. Both PE cohorts had higher SV measurements by Doppler than SV by MOD (with or without indexing by BSA). Mean SV measurements, irrespective of measurement approach, were statistically reduced in patients who experienced clinical deterioration vs those who did not.

**Table 2.** Bivariable analysis of echocardiographic measurements compared by primary outcome.^*^

Echocardiographic measurements	Primary outcome = YES (N = 39)	Primary outcome = NO (N = 331)	Overall (N = 370)	*P*-value
**Internal diameter of LVOT (cm)**				
Mean (SD)	2.1 (0.2)	2.1 (0.2)	2.1 (0.3)	0.63
**Velocity time integral at LVOT (cm)**				
Mean (SD)	13.6 (8.0)	17.9 (6.0)	17.5 (6.3)	0.003
**Stroke volume as determined at LVOT (mL)**				
Mean (SD)	41.7 (28.0)	57.2 (27.0)	55.7 (27.4)	0.004
Missing	10 (25.6%)	57 (17.3%)	69 (18.6%)	
**Stroke Volume Index at LVOT (mL/m^2^)**				
Mean (SD)	21.2 (13.4)	27.5 (12.2)	26.9 (12.4)	0.001
**Cardiac output as determined at LVOT (mL/min)**				
Mean (SD)	3860 (2290)	4890 (2150)	4790 (2180)	0.02
Missing	10 (25.6%)	57 (17.3%)	69 (18.6%)	
**Cardiac output index as determined at LVOT (mL/min/m^2^)**				
Mean (SD)	1970 (1100)	2340 (953)	2300 (972)	0.05
**Stroke volume, by MOD (mL)**				
Mean (SD)	36.2 (15.8)	49.9 (20.1)	48.4 (20.1)	< .001
Missing	0 (0%)	11 (3.3%)	11 (3.0%)	
**Stroke Volume index by MOD, (mL/m^2^)**				
Mean (SD)	18.8 (8.7)	24.0 (8.9)	23.4 (9.0)	0.001
**Cardiac output, by MOD (mL/min)**				
Mean (SD)	3460 (1310)	4320 (1760)	4230 (1740)	0.003
Missing	0 (0%)	11 (3.3%)	11 (3.0%)	
**Cardiac Output Index by MOD (mL/min/m^2^)**				
Mean (SD)	1760 (734)	2070 (741)	2040 (746)	0.02
**Severity of mitral regurgitation, if present**				
None	20 (51.3%)	170 (52%)	192 (51.9%)	0.36
Mild	14 (35.9%)	123 (37.2%)	137 (37.0%)	
Moderate	1 (2.6%)	10 (3.0%)	11 (3.0%)	
Severe	1 (2.6%)	1 (0.3%)	2 (0.5%)	
Missing	3 (7.7%)	25 (7.6%)	28 (7.6%)	
**LV basal width (cm)**				
Mean (SD)	4.0 (0.9)	4.2 (0.8)	4.2 (0.8)	0.07
Missing	0 (0%)	6 (1.8%)	6 (1.6%)	
**LV ejection fraction, estimated (%)**				
Mean (SD)	53.9 (14.7)	54.7 (10.9)	54.6 (11.4)	0.66
Missing	0 (0%)	2 (0.6%)	2 (0.5%)	
**RV basal width (cm)**				
Mean (SD)	4.3 (0.9)	4.2 (0.8)	4.3 (0.8)	0.87
Missing	2 (5.1%)	13 (4.0%)	15 (4.1%)	
**RV:LV basal width ratio**				
Mean (SD)	1.1 (0.3)	1.1 (0.3)	1.1 (0.3)	0.09
Missing	2 (5.1%)	18 (5.4%)	20 (5.4%)	
**RV mid width (cm)**				
Mean (SD)	3.6 (0.9)	3.7 (2.6)	3.7 (2.5)	0.80
Missing	4 (10.3%)	22 (6.6%)	26 (7.0%)	
**RV major length (cm)**				
Mean (SD)	7.0 (0.9)	7.2 (3.7)	7.2 (3.5)	0.68
Missing	5 (12.8%)	31 (9.4%)	36 (9.7%)	
**Peak tricuspid regurgitant jet velocity (m/s)**				
Mean (SD)	2.9 (0.6)	2.9 (0.7)	2.9 (0.7)	0.15
Missing	15 (38.5%)	92 (28.0%)	107 (28.9%)	
**TAPSE (cm)**				
Mean (SD)	1.5 (0.5)	1.8 (0.5)	1.8 (0.5)	0.003
Missing	8 (20.5%)	52 (15.7%)	60 (16.2%)	
**RV annulus peak systolic velocity S' (cm/s)**				
Mean (SD)	10.7 (5.2)	11.5 (4.4)	11.4 (4.5)	0.37
Missing	10 (25.6%)	68 (20.5%)	78 (21.1%)	
**Initial high-sensitivity troponin (ng/L)**				
Mean (SD)	237 (332)	175 (378)	182 (374)	0.34
Missing	2 (5.1%)	4 (1.2%)	6 (1.6%)	
**Initial BNP level (pg/mL)**				
Mean (SD)	435 (661)	290 (387)	304 (424)	0.05
Missing	3 (7.7%)	14 (4.2%)	17 (4.6%)	

* Normal Values are provided for comparison: The World Alliance of Societies of Echocardiography Study^19^ published normal values for two echocardiographic assessments (Doppler and MOD) for variables in the calculation of cardiac output for adult subjects without diseases. By Doppler, normal values are velocity time integral 20.2 ± 3.6 mm, stroke volume 68.7 ± 17.0 ml, SV indexed by body surface area 38.7 ± 8.1 ml/m^2^, cardiac output 4.58 ± 1.12 L/min/m^2^, and cardiac index 2.6 ± 0.58 L/min/m^2^. By two-dimensional echocardiography, normal values are: SV 58.4 ± 15.4 ml, SV indexed 32.7 ± 6.8 ml/m^2^, cardiac output 3.88 ± 1.00 L/min, and cardiac index 2.18 ± 0.48 L/min/m^2^.

The American Society of Echocardiography^16^ reports the following values as abnormal: RV basal diameter > 4.2 cm, TAPSE < 1.6 cm, pulse Doppler peak velocity, S’ < 10 cm/s.

*LVOT*, left ventricular outflow tract; *MOD*, method of discs; *LV*, left ventricle; *RV*, right ventricle; *TAPSE*, tricuspid annular planar systolic excursion; *BNP*, brain natriuretic peptide.

Table 3 shows results from the LASSO model that started with all SV and CO measures considered. It ended with selecting only SV by MOD, among other patient and clinical characteristics that were also predictive. For imputed values, the best predictor was SV by MOD with OR 0.72 (CI 0.53, 0.94; *P* = 0.02). As SV increased, the probability of primary outcome decreased. Recent hospitalization and metastatic solid tumor were other independent predictors. SV Doppler, TAPSE, and RV basal width had non-significant ORs. The SV by MOD was more strongly associated with the primary outcome than SV Doppler. The OR of 0.72 for SV MOD implies that for every 10 mL increase in SV, there was 28% decreased odds of the primary outcome. That is, person A with SV of 60 mL had 0.72 times the odds of the outcome relative to person B with an SV of 50 mL (ie, 1.0 – 0.72 = 0.28).

**Table 3.** Least absolute shrinkage and selection operator (LASSO) regression results using imputed values.

Predictors	Primary composite outcome		
	*Odds ratios*	*Confidence interval*	*P*-value
Stroke volume as determined at MOD (mL)	0.72	0.52–0.98	0.04
Lowest systolic blood pressure within 3 hours of presentation (mmHg)	0.98	0.96–1.00	0.02
Lowest oxygen saturation within 3 hours, %	0.95	0.91–1.00	0.06
Highest respiratory rate within 3 hours (breaths per minute)	1.03	0.99–1.07	0.09
Initial heart rate (beats per minute)	1.01	0.99–1.04	0.35
Velocity time integral determined at LVOT (cm)	0.96	0.87–1.05	0.38
Metastatic solid tumor: Yes	3.32	1.16–9.03	0.02
Recent hospitalization: Yes	4.68	1.87–11.65	< .001
Observations	370		
R^2^ Tjur	0.265		

*LVOT*, left ventricular outflow tract; *MOD*, method of discs; *RV*, right ventricle; *TAPSE*, tricuspid annular planar systolic excursion.

Table 4 shows Youden’s index of the optimal cut-off values for TTE indices to maximize sensitivity and specificity. The most significant predictors were SV MOD, VTI, and SV Doppler, with best predictive performance for acute clinical deterioration in terms of balance between sensitivity and specificity. For common metrics of RV size and systolic function, highest AUC was the TAPSE cut-off.

**Table 4.** Prognostic performance of optimal echocardiography cut-off points for the primary outcome.

Variable	Cut-off point	*P*-value	Sensitivity	Specificity	PPV	NPV	AUC	Odds ratio
Internal diameter of LVOT	2.0	0.63	84 (68, 100)	23 (17, 29)	10 (5, 14)	94 (87, 100)	0.54 (0.4, 0.7)	1.6 (0.5, 5.7)
RV major length (cm)	6.3	0.69	85 (73, 97)	26 (21, 31)	12 (8, 16)	94 (89, 99)	0.52 (0.4, 0.6)	2.1 (0.8, 5.5)
RV basal width (cm)	4.9	0.88	24 (1, 38)	85 (81, 89)	16 (6, 26)	91 (87, 94)	0.51 (0.4, 0.6)	1.8 (0.8, 4.2)
RV mid width (cm)	4.5	0.80	43 (26, 59)	65 (60, 70)	12 (6, 18)	91 (87, 95)	0.49 (0.4, 0.6)	1.4 (0.7, 2.9)
LV basal width (cm)	5.4	0.07	51 (36, 67)	74 (69, 78)	19 (12, 27)	93 (89, 96)	0.63 (0.5, 0.7)	3.0 (1.5, 5.8)
RV: LV basal width ratio	1.0	0.08	65 (49, 80)	55 (50, 61)	15 (9, 20)	93 (89, 97)	0.58 (0.5, 0.7)	2.3 (1.1, 4.7)
Peak tricuspid regurgitant jet velocity (m/s)	3.2	0.63	67 (48, 86)	45 (38, 51)	11 (6, 16)	93 (88, 98)	0.53 (0.4, 0.7)	1.6 (0.7, 3.9)
LV ejection fraction, estimated (%)	55.0	0.66	21 (8, 33)	87 (84, 91)	16 (6, 26)	90 (87, 93)	0.48 (0.4, 0.6)	1.8 (0.8, 4.1)
TAPSE (cm)	1.8	0.00	81 (67, 95)	47 (41, 53)	15 (9, 20)	96 (92, 99)	0.67 (0.6, 0.8)	3.7 (1.5, 9.4)
RV annulus peak systolic velocity S’ (cm/s)	16.10	0.35	34 (17, 52)	88 (84, 92)	24 (11, 38)	92 (89, 96)	0.59 (0.5, 0.7)	3.9 (1.7, 9.2)
Stroke volume, by MOD (mL)	54.3	0.00	69 (55, 84)	66 (61, 71)	20 (13, 27)	95 (92, 98)	0.72 (0.6, 0.8)	4.3 (2.1, 8.9)
Stroke volume indexed by BSA, by MOD (mL/m^2^)	27.2	0.02	57 (36, 77)	75 (7, 80)	14 (7, 22)	96 (93, 98)	0.65 (0.5, 0.8)	3.9 (1.6, 9.2)
Cardiac output, by MOD (mL/min)	5,916	0.00	36 (21, 51)	86 (83, 90)	25 (13, 36)	92 (89, 95)	0.64 (0.6, 0.7)	3.6 (1.7, 7.4)
Cardiac output indexed by MOD, mL/min/m^2^	2,820.7	0.19	39 (19, 59)	86 (82, 90)	17 (7, 28)	95 (92, 98)	0.58 (0.4, 0.7)	4.0 (1.6, 9.7)
Velocity time integral at LVOT, cm	19.0	0.00	76 (58, 94)	67 (60, 74)	21 (12, 30)	96 (93, 99)	0.72 (0.6, 0.8)	6.7 (2.3, 18.8)
Velocity time integral, indexed by BSA, cm/m^2^	13.1	0.28	33 (7, 60)	90 (86, 95)	19 (2, 36)	95 (92, 98)	0.60 (0.4, 0.8)	4.6 (1.2, 16.7)
Stroke volume as determined at LVOT (mL)	73.0	0.00	72 (56, 89)	67 (61, 73)	19 (12, 26)	96 (93, 99)	0.70 (0.6, 0.8)	5.3 (2.3, 12.5)
Stroke volume indexed, at LVOT, ml/m^2^	37.4	0.06	47 (25, 70)	86 (82, 90)	19 (8, 30)	96 (94, 98)	0.64 (0.5, 0.8)	5.6 (2.1, 14.6)
Cardiac output as determined at LVOT (mL/min)	4,284	0.02	79 (65, 94)	47 (41, 53)	14 (9, 19)	96 (92, 99)	0.66 (0.6, 0.8)	3.5 (1.4, 8.8)
Cardiac output indexed at LVOT (mL/min/m^2^)	3,022	0.23	47 (25, 70)	81 (76, 85)	14 (6, 23)	96 (93, 98)	0.59 (0.4, 0.8)	3.78 (1.5, 9.8)

*PPV*, positive predictive value; *NPV*, negative predictive value; *AUC*, area under the curve; *LV*, left ventricle; *RV*, right ventricle; *MOD*, method of discs; *LVOT*, left ventricular outflow tract; *TAPSE*, tricuspid annular planar systolic excursion.

The RF model determined independent predictors of our primary outcome and generated a variable importance plot (Supplemental Figure). The SV by MOD, VTI, and CO by MOD were the highest ranking TTE predictors for the primary outcome. Performance metrics for LASSO and RF models included AUC 0.8 and Brier score 0.08 (Table 5). Calibration and decision-curve analysis plots are included in the Appendix.

**Table 5.** Performance and calibration of prediction models.^*^

Model	Discrimination	Calibration			
	Sensitivity vs 1- specificity plot AUC (95% CI)	Brier score	Scaled Brier	Calibration intercept	Calibration slope
Logistic model^**^	0.81 (0.69, 0.92)	0.08 (0.06, 0.10)	0.17 (0, 0.36)	0.02 (−0.54, 0.51)	0.83 (0.37, 1.59)
Random forest^†^	0.79 (0.71, 0.85)	0.08	0.15	−0.08 (−0.44, 0.27)	1.12 (0.73, 1.51)

* Using a scale of 0 to 1, indicators of better performance metrics are: AUC (closer to 1), Brier score (lower), scaled Brier (closer to zero), calibration intercept (closer to zero), calibration slope (closer to 1).^26^

** Due to issues of collinearity, LASSO regression was for variable selection based on 10-fold cross validation and selecting variables based on the lambda minimum. For the LASSO selected variables, we used Monte Carlo cross validation across 500 iterations with a 70/30 split between training and test data to fit repeated logistic models for the primary outcome. To assess discrimination, performance, and calibration, we reported the averages across iterations and 95% coverage intervals (ie, the 2.5th and 97.5th quantile from the 500 iterations).

† For comparison to the random forest (RF) fitted model, we estimated the same metrics based on out-of-bag samples from the RF fitted model, and calibration plot based on out-of-bag predicted probability estimates.

*AUC*, area under the receiver operating curve; *CI*, confidence interval.

Table 6 shows patients who received escalated interventions had significantly lower SV or surrogate measurements, greater RV dilatation, and lower (worse) RV systolic function than patients who received anticoagulation monotherapy.

**Table 6.** Bivariable analysis of echocardiographic measurements compared by treatment group.^*^

Echocardiographic measurements	Immediate or delayed escalated PE interventions (N = 56)	Anticoagulation monotherapy watch and wait (N = 314)	Mean difference (95% confidence interval)	*P*-value
Velocity time integral at LVOT, cm				
Mean (SD)	14.0 (7.4) n = 27	18.0 (6.0) n = 179	−3.9 (−6.5, −1.4)	0.002
Velocity time integral LVOT indexed by BSA, cm/m^2^				
Mean (SD)	6.7 (4.0) n = 27	8.9 (3.5) n = 156	−2.2 (−3.9, −0.5)	0.004
Stroke volume as determined at LVOT (mL)				
Mean (SD)	44.4 (23.4) n = 46	57.8 (27.6) n = 255	−13.3 (−21.6, −4.8)	0.002
Stroke volume as determined at LVOT indexed by BSA, mL/m^2^				
Mean (SD)	21.3 (11.3) n = 46	27.9 (12.3) n = 255	−6.60 (−10.2, −2.9)	<0.001
Stroke volume, by MOD (mL)				
Mean (SD)	39.3 (16.3) n = 55	50.1 (20.3) n = 304	−10.8 (−16.4, −5.1)	<0.001
Stroke volume, by MOD indexed by BSA, mL/m^2^				
Mean (SD)	19.0 (7.4) n = 52	24.3 (9.0) n = 278	−5.30 (−7.6, −3.0)	<0.001
RV basal width (cm)				
Mean (SD)	4.6 (0.8) n = 54	4.2 (0.8) n = 301	0.4 (0.2, 0.7)	<0.001
RV: LV basal width ratio				
Mean (SD)	1.2 (0.3) n = 52	1.0 (0.3) n = 298	0.2 (0.13, 0.29)	<0.001
TAPSE (cm)				
Mean (SD)	1.4 (0.4) n = 45	1.8 (0.5) n = 265	−0.4 (−0.6, −0.3)	<0.001
RV annulus peak systolic velocity S’ (cm/s)				
Mean (SD)	9.3 (2.8) n = 44	11.8 (4.6) n = 248	−2.5 (−3.9, −1.1)	0.001

* Normal values are provided for comparison: The World Alliance of Societies of Echocardiography Study^19^ published normal values for two echocardiographic assessments (Doppler and MOD) for variables in the calculation of cardiac output for adult subjects without diseases. By Doppler, normal values are velocity time integral 20.2 ± 3.6 mm, stroke volume 68.7 ± 17.0 ml, SV indexed by body surface area 38.7 ± 8.1 ml/m^2^, cardiac output 4.58 ± 1.12) L/min/m^2^ and cardiac index 2.6 ± 0.58 L/min/m^2^. By two-dimensional echocardiography, normal values are: SV 58.4 ± 15.4 ml, SV indexed 32.7 ± 6.8 ml/m^2^, cardiac output 3.88 ± 1.00 L/min and cardiac index 2.18 ± 0.48 L/min/m^2^.

The American Society of Echocardiography^16^ reports the following values as abnormal: RV basal diameter >4.2 cm, TAPSE <1.6 cm, pulse Doppler peak velocity, S’ <10 cm/s.

*PE*, pulmonary embolism; *BSA*, body surface area; *LVOT*, left ventricular outflow tract; *MOD*, method of discs; *LV*, left ventricle; *RV*, right ventricle; *TAPSE*, tricuspid annular planar systolic excursion.

## DISCUSSION

In our cohort of 370 intermediate-risk patients identified in the ED, both early TTE metrics for SV were strongly associated with acute clinical deterioration. By both bivariable and multivariable analyses, TTE metrics for SV indices and RV systolic function were better predictors of the primary outcome than RV size or troponin levels. The two methods of measuring SV were correlated but not interchangeable. Echocardiographic parameters (SV by MOD, VTI, CO by MOD, and SV LVOT) were identified among the 20 highest ranking predictors of all candidate variables for the primary outcome. Intermediate-risk patients subsequently treated with escalated interventions had significantly larger basal RV size, lower RV systolic function (TAPSE and S’wave), and lower SV parameters (VTI, SV MOD, and SV Doppler) than those treated with anticoagulation monotherapy. Even with tradeoffs and limitations of determining optimal cut-off values on combined sensitivity and specificity (Table 4), SV, VTI, and CO predictors had the best predictive ability. Optimal cut-offs shown in Table 4 may discriminate between patients at risk of subsequent deterioration vs those at low risk. High NPVs among these metrics would suggest low-risk patients were correctly identified.

Our cohort had lower mean SV than normal values for healthy adults. The World Alliance of Societies of Echocardiography identified normal mean SV in adults as VTI 20.2 ± 3.6 centimeters (cm), SV Doppler 68.7 ± 17.0 mL, and SV MOD 58.4 ± 15.4 mL.^19^ Means for our cohort were: VTI 17.5 ± 6.3 cm, SV Doppler 55.7 ± 27.4 mL, and SV MOD 48.4 ± 20.1 mL. Mean SV was even lower for our patients who had primary outcome (VTI 13.6, SV Doppler 41.7, SV MOD 36.2 mL).

The strength of this study is identification of a possible predictor with a plausible physiological mechanism for acute clinical deterioration that has been minimally reported in the PE medical literature. Abrupt arterial occlusion on PE may lead to increased RV afterload. Worsening PE-provoked physiology involves key steps of decreased RV systolic function, reduced RV output, LV underfilling, reduced LV CO, decreased blood pressure, and reduced RV perfusion and oxygen delivery before obstructive shock and death.^5^^,^^29^ Although it is premature to determine causality of single SV metrics, reduced LV CO and its surrogates (SV and VTI) represent an advanced stage on the pathway toward hemodynamic instability or death from acute PE.^5^^,^^29^ In patients with RV dilatation, low SV might suggest subclinical shock, inadequate LV filling and output, and suggest this patient be treated as ***intermediate-high*** ***risk***. Thus, SV may identify a subgroup of intermediate-risk patients with a more favorable risk profile for 11escalated interventions.^30^

Although bivariable and multivariable analyses showed mean vital signs were associated with the outcome-positive group (eg, lowest SBP, highest heart rate, and highest respiratory rate), the mean values themselves did not lead to reassignment from intermediate risk to high risk; they merely disqualified patients from being considered low risk by PE severity index (PESI)/simplified PESI (sPESI).^31^^,^^32^ At presentation, our patients were without cardiac arrest, obstructive shock, or persistent hypotension and thus were not classified as high risk by ESC criteria despite having higher heart rates and lower SBP (<100 mm Hg but >90 mm Hg).^5^ In normotensive PE patients, we believe lower SV measurements provide more information about subclinical or impending shock in more severe cases than RV dilatation alone.

Existing PE studies that report SV use various techniques, outcomes, and timepoints. Few report SV being predictive of clinical deterioration when intermediate risk is defined by presence of RV abnormalities. Some studied CO surrogates using RV outflow tract or LV CO, or combined RV pressure assessments with LV SV assessments.^18^^,^^20^^,^^21^^,^^33^^,^^34^ For example, Kamran et al studied 343 PE patients evaluated by a PERT, who had pulmonary artery systolic pressure (PASP) and LV outflow tract SV measurements.^34^ A PASP/SV ratio ≥1.0 mm Hg/mL was associated with an increased risk of their primary outcome (death, cardiac arrest, and escalated interventions).

We and other researchers argue that RV dilatation is insufficient to distinguish which intermediate-risk PE patient is suffering from a low-flow state and likely to experience clinical deterioration.^8^^,^^9^^,^^11^ While a meta-analysis concluded RV parameters were associated with poor clinical outcomes, the authors cautioned of methodological issues with low-quality evidence for most included studies.^12^ Also, RV dysfunction definitions vary, and TTE measurement thresholds are not commonly incorporated into decision-making for intermediate-risk PE patients.^8^^,^^12^ In this study, SV had greater prognostic value than RV size or troponin in distinguishing the transition to hemodynamic or clinical instability.

A retrospective study of intermediate-risk PE patients by Prosperi-Porta et al reported superior performance of SV index over RV measurements for anticipating PE-related adverse events (similar to our primary outcome).^18^ Unlike our study, they included patients without RV abnormalities because they defined intermediate risk as sPESI >zero. Their cohort had lower acuity overall than ours. In contrast, our definition of intermediate risk included abnormal RV by CT or elevated cardiac biomarkers. Given our cohort had higher severity, our challenge was to identify unique predictors among patients with PE-associated cardiac dysfunction. Our outcome event rate (10.5%) was more than twice that reported by Prosperi-Porta et al.

Yuriditsky et al used VTI measured at the LVOT as an SV surrogate and defined low VTI as <15 cm.^20^ Patients who died or had cardiac arrest had lower mean VTI than patients who did not (13.4 [3.9] and 18.3 [5.0] cm, respectively). Patients who experienced shock or needed reperfusion had lower mean VTI than those who did not (12.8 [3.2] and 18.6 [4.8] cm, respectively). Babes et al studied normotensive patients with PE and RV:LV of ≥1 and showed VTI <15 cm had PPV and NPV of 75% and 95%, respectively, for clinical deterioration.^35^ We had similar findings.

In our study, patients with the primary outcome had lower mean VTI than those who did not (13.6 [8.0] and 17.9 [6.0] cm, respectively). Patients who received escalated interventions had lower mean VTI than those who did not (13.96 [7.4] and 17.9 [6.0] cm, respectively). In our study, intermediate-risk patients who received escalated interventions had lower VTI, SV, and RV systolic function and larger RV chambers than adults without disease.^16^^,^^19^^,^^36^ Patients who concerned clinicians enough to receive escalated interventions had significantly lower SV and VTI, greater RV dilation, and lower (worse) RV systolic function than patients treated with anticoagulation monotherapy. We believe these differences identify the subgroup of patients with current or impending subclinical shock.

The clinical relevance of our study findings is that SV measurements may be used to 1) identify a subgroup of intermediate-risk patients at increased risk for clinical deterioration, and 2) determine candidacy for escalated interventions. The SV can be easily measured, incorporated into clinical practice, and used to inform prompt treatment with escalated interventions for intermediate-high risk PE patients in the ED. Ultrasound use is integral to training and practice of emergency medicine (EM) and is a required skillset of physicians certified by the American Board of Emergency Medicine. The near future involves more emergency clinicians acquiring and using clinically indicated ultrasound.^37^ In addition, automated VTI, SV, and CO measurements are emerging in point-of-care cardiac ultrasound applications by vendors and becoming available to clinicians with basic/intermediate advanced cardiac ultrasound skills.^38^

Given the knowledge gap in RV failure research, this study supports further investigation into the impact of SV on clinical outcomes and decision-making.^11^^,^^30^ Future studies may be designed to include SV as a predictor or include changes in SV as an efficacy outcome of PE interventions. Such reports may provide evidence to support or refute the use of SV metrics to indicate candidacy for escalated interventions or inform decision-making in EDs, including the need to provide intensive care or transfer to a healthcare facility with a PERT. The end result may be inclusion of SV in risk stratification tools used by PERTs.

## LIMITATIONS

First, we did not report on aortic insufficiency as a confounder of LVOT VTI and SV Doppler measurements. The SV Doppler assessments will be limited by outflow tract obstruction and measurements affected by conditions such as hypertrophic cardiomyopathy and hypovolemia. Accuracy of SV assessment may be affected by dysrhythmias and underestimation of forward flow by aortic and mitral valvular insufficiency. Second, treatment teams were not blinded to TTE results. However, most were agnostic to the hypothesized clinical significance of the measurements. It is unlikely treating physicians incorporate metrics on RV size, systolic function, pressure, and SV in their clinical decision-making. There are no established thresholds for TTE metrics or recommendations to trigger early use of escalated interventions. Third, we did not perform inter-rater reliability measures. Finally, although discrimination and calibration metrics show SV as a predictor of clinical deterioration, there was no external validation to further address usefulness and impact.

## CONCLUSION

Echocardiographic hemodynamic parameters were among the best predictors of clinical deterioration. Low stroke volume preceded and predicted clinical deterioration. Lower SV was found in patients treated with escalated intervention than in those without. We recommend further inquiry into incorporating SV into pulmonary embolism risk stratification, prognosis, and decisions on patient disposition and clinical management.

## Supplementary Information




