# Supplementary material for: Low Stroke Volume Predicts Deterioration in Intermediate-Risk Pulmonary Embolism: Prospective Study
Source: West J Emerg Med. 2024 Jun 14;25(4):533–47. doi: 10.5811/westjem.18434 (PMC11254154; doi:10.5811/westjem.18434)
Supplement: Supplementary file 1 [file wjem-25-533-s001.pdf]

Figure S1: Variable importance plot for predicting primary outcome*

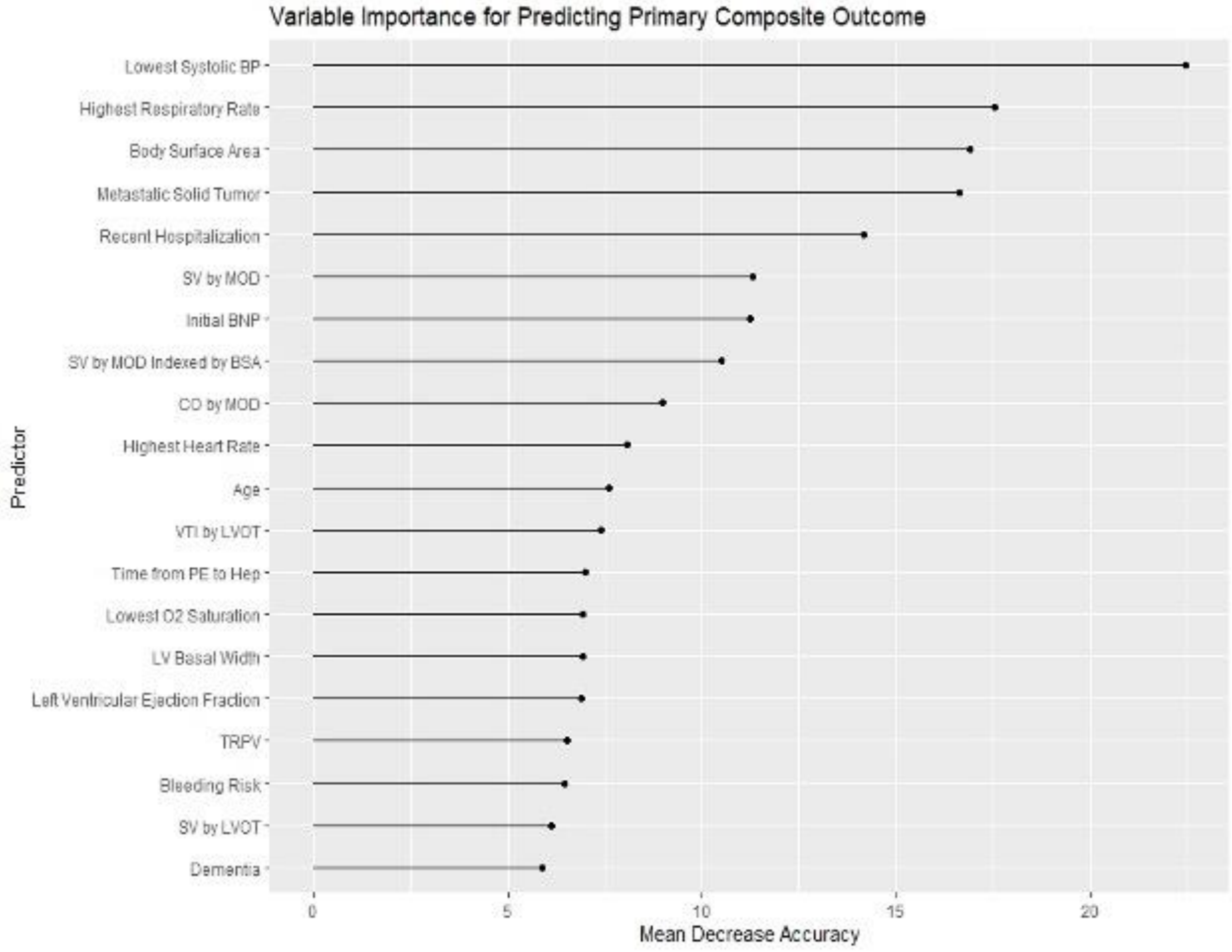

* Abbreviations: BP = blood pressure, SV = stroke volume, MOD = method of discs, BNP = brain natriuretic peptide, BSA = body surface area, CO = cardiac output, VTI = velocity time integral, LVOT= left ventricular outflow tract, LV = left ventricle, TRPV = tricuspid regurgitant peak velocity
