# Supplementary material for: Low Stroke Volume Predicts Deterioration in Intermediate-Risk Pulmonary Embolism: Prospective Study
Source: West J Emerg Med. 2024 Jun 14;25(4):533–47. doi: 10.5811/westjem.18434 (PMC11254154; doi:10.5811/westjem.18434)
Supplement: Supplementary file 2 [file wjem-25-533-s002.pdf]

APPENDIX

The following two figures show calibration plots (**Figure S2**) and decision curve analysis plots (**Figure S3**) for LASSO and random forest models.

Figure S2: Calibration plot for logistic regression and random forest models

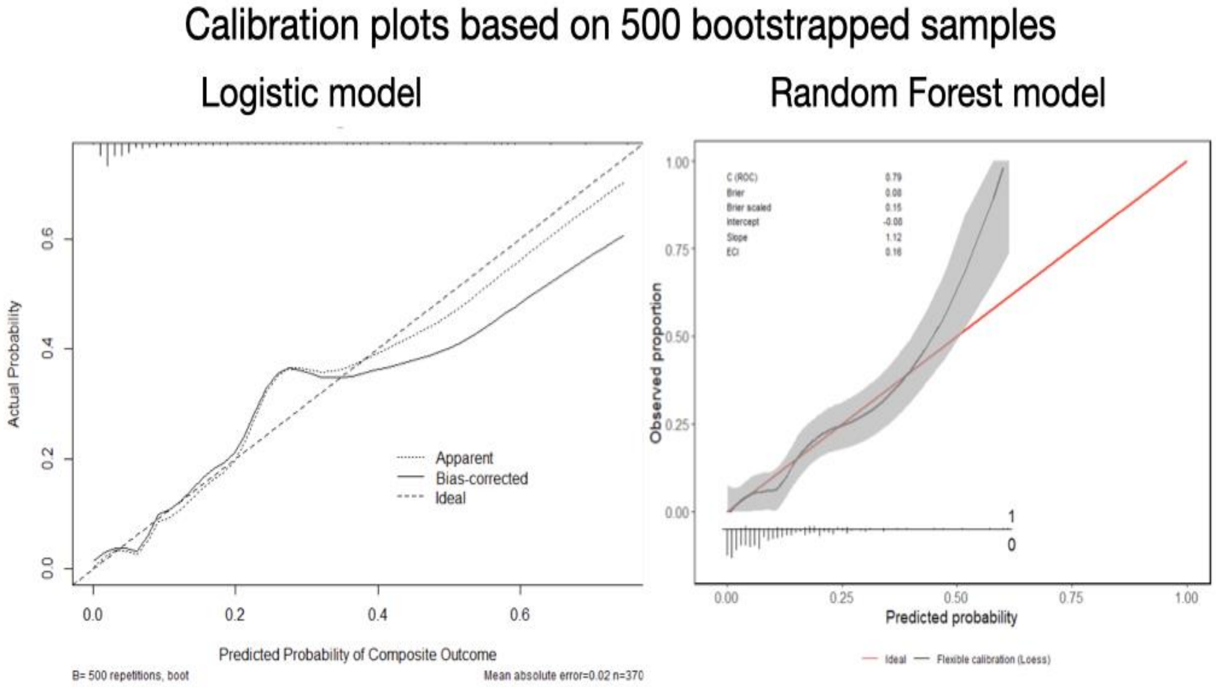

** Calibration plot from 500 bootstrapped samples to assess model calibration visually.*

Figure S3: Net benefit of logistic regression and random forest models based on decision curve analysis*

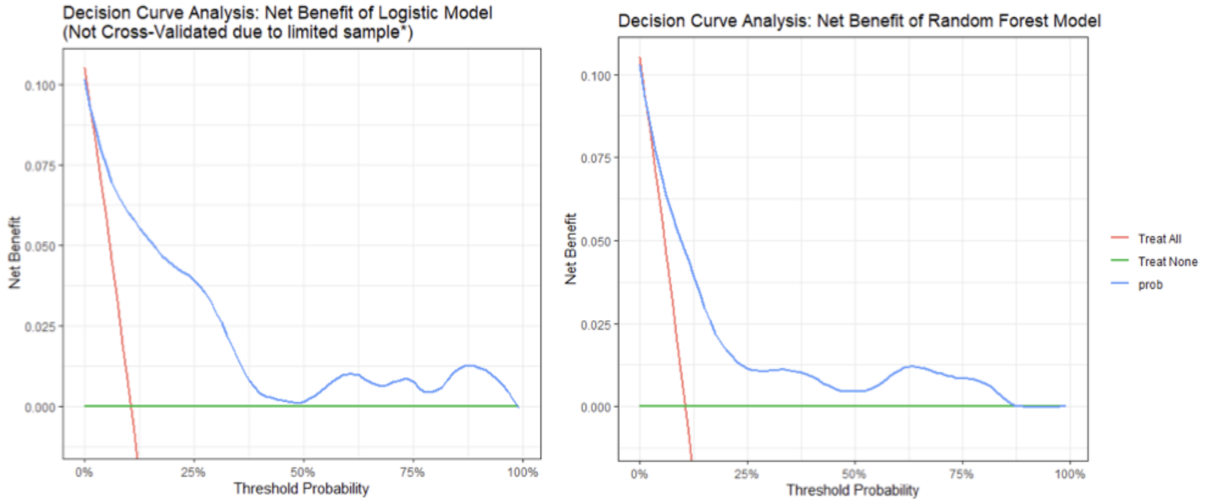

Legend: The figures show net benefit for the logistic model (left) and the random forest model (right). Benefit (Y axis) is shown across a range of preferences (X axis). Net benefit (blue line) in the right upper quadrant depicts the net positive benefit consequences in classification of the decisions made on the basis of a predictive model versus default approach options. One default approach assumes all will have the primary outcome and take resources or measures to intervene (red line). The alternative default approach assumes no patients will have the primary outcome and providers do nothing (green line). The net benefit demonstrates at least some clinical utility in discriminate between predicted high and low risk patients, and act accordingly based on their classification.[37]

* As discussed by Steyerberg et al., we note that discrimination (as measured by area under the receiver operating curve) and calibration in terms of accuracy of predicted probabilities do not necessarily translate to clinical utility.[27] Machine learning models, such as random forest, will often have poor calibration in the presence of unbalanced

data. Thus, we demonstrate clinical utility via net benefit based on decision curve analysis.[28]
