# Supplementary material for: Low Stroke Volume Predicts Deterioration in Intermediate-Risk Pulmonary Embolism: Prospective Study
Source: West J Emerg Med. 2024 Jun 14;25(4):533–47. doi: 10.5811/westjem.18434 (PMC11254154; doi:10.5811/westjem.18434)
Supplement: Supplementary file 3 [file wjem-25-533-s003.docx]

Appendix 1. List of variables for the Classification And Regression Tree (CART) analysis

Age

NIHSS at CCRU arrival

Intravenous thrombolytics (IV tPA)

Time intervals

- ED in-out
- CCRU arrival to angiography suite
- IR to groin puncture
- groin puncture to recanalization

Laboratory values

- Serum creatinine
- Serum sodium
- INR

Types of vessel occlusion

TICI score
